# A Novel Hexavalent Capsular Polysaccharide Conjugate Vaccine (GBS6) for the Prevention of Neonatal Group B Streptococcal Infections by Maternal Immunization

**DOI:** 10.1093/infdis/jiz062

**Published:** 2019-02-19

**Authors:** Ed T Buurman, Yekaterina Timofeyeva, Jianxin Gu, Jin-hwan Kim, Srinivas Kodali, Yongdong Liu, Terri Mininni, Soraya Moghazeh, Danka Pavliakova, Christine Singer, Suddham Singh, Luke D Handke, Jason Lotvin, A Krishna Prasad, Ingrid L Scully, Robert G K Donald, Kathrin U Jansen, Annaliesa S Anderson

**Affiliations:** Vaccine Research and Development, Pfizer, Pearl River, New York

**Keywords:** GBS6, capsular polysaccharide, *Streptococcus agalactiae*, group B streptococcus, maternal vaccine, conjugate, CRM_197_

## Abstract

**Background:**

Group B streptococcus (GBS) causes serious diseases in newborn infants, often resulting in lifelong neurologic impairments or death. Prophylactic vaccination of pregnant women prior to delivery could provide comprehensive protection, as early onset and late-onset disease and maternal complications potentially could be addressed.

**Methods:**

Capsular polysaccharide conjugate vaccine GBS6 was designed using surveillance data yielded by whole-genome sequencing of a global collection of recently recovered GBS isolates responsible for invasive neonatal GBS disease. Capsular polysaccharides were isolated, oxidized using sodium periodate, and conjugated to CRM_197_ by reductive amination in dimethyl sulfoxide. Immune responses in mice and rhesus macaques were measured in a multiplex Luminex immunoglobulin G (IgG) assay and opsonophagocytic activity assays.

**Results:**

The optimized conjugates were immunogenic, alone and in combination, in mice and rhesus macaques, inducing IgG antibodies that mediated opsonophagocytic killing. Active immunization of murine dams with GBS6 prior to mating resulted in serotype-specific protection of pups from a lethal challenge with GBS. Protection following passive administration of serotype-specific IgG monoclonal antibodies to dams demonstrated conclusively that anticapsular polysaccharide IgG alone is sufficient for protection.

**Conclusions:**

The findings support the ongoing clinical evaluation of maternal GBS6 vaccination as a potential alternative method to prevent GBS disease in infants.

## Teaser

Six-valent capsular polysaccharide conjugate vaccine GBS6 for the prevention of neonatal Group B Streptococcal (GBS) infections was evaluated in preclinical models. Vaccination with GBS6 or administration of serotype-specific IgG to pregnant dams protected pups against lethal challenges with GBS.

*Streptococcus agalactiae*, or group B streptococcus (GBS), is an encapsulated gram-positive opportunistic pathogen associated with lower intestinal and rectovaginal colonization in 11%–35% of women [1]. In pregnant women, the species causes ascending infections ranging from relatively benign urinary tract infections to chorioamnionitis, potentially resulting in stillbirth, preterm delivery, and puerperal sepsis. In infants, the disease most commonly occurs within the first week of life (hereafter, “early onset disease”) and less commonly occurs between 7 and 90 days of life (hereafter, “late-onset disease”). Neonatal GBS disease generally manifests as sepsis, pneumonia, and meningitis. The global mortality rate is 8.4%, with rates in the United States and Africa of 7% and 19%, respectively [2]. Infants surviving GBS meningitis can experience severe neurological consequences, including cognitive delay, cerebral palsy, blindness, or hearing loss [3].

In the United States, universal screening for rectovaginal GBS colonization is recommended for pregnant women between 35 and 37 weeks of gestation, and carriers receive intrapartum antibiotic prophylaxis during delivery, to prevent disease [4]. After introduction of intrapartum antibiotic prophylaxis, the incidence of early onset disease decreased from 1.7 to 0.22 cases/1000 live births. Late-onset disease rates, however, have remained unaffected, at 0.25 cases/1000 live births [4, 5]. In addition, intrapartum antibiotic prophylaxis will not affect GBS infections before delivery, either in pregnant women or the developing fetus. Furthermore, as prophylaxis to prevent GBS diseases and infections following cesarean section has led to administration of antibiotics in approximately 40% of all labor and delivery procedures in the United States, apprehension about overuse of antibiotics is growing because of the propensity for development of antimicrobial resistance [6]. Particularly concerning is exposure to antibiotics when the neonate’s microbiome becomes established, which may lead to health consequences later in life [7]. Finally, implementation of prenatal screening and intrapartum antibiotic prophylaxis requires a well-developed infrastructure that is not available in most low-to-middle-income countries, where healthcare access and standards of prenatal care vary greatly.

GBS express capsular polysaccharides (CPs) as a mechanism of immune evasion [8]. The CPs contain terminal sialic acid moieties, impeding binding of the complement component C3 and thus shielding the pathogen from the immune system. Low maternal serotype III–specific immunoglobulin G (IgG) concentrations among women colonized with GBS serotype III have been linked with infant susceptibility to early onset disease due to serotype III strains [9], with the association later extended to include serotypes Ia and V [10, 11]. Because IgG is actively transported across the placenta from mother to baby during the last weeks of pregnancy [12], elevated anti-CP IgG levels in the mother can ensure protective IgG antibody levels in the fetus and newborn baby. Most women of childbearing age will have been colonized at various times in their lives before becoming pregnant [1]. Thus, their immune response should have been primed to recognize GBS CP, and administration of a single vaccine dose may be sufficient to induce an anamnestic protective response. Early clinical testing showed that unconjugated CP was poorly immunogenic; CP conjugated to carrier protein, however, proved to be highly immunogenic in clinical studies [13]. GBS3, a trivalent GBS CP conjugate vaccine advanced to phase 2 clinical trials [14], but development was stopped after its limited disease coverage emerged [15]. Here, we describe the preclinical evaluation of GBS6, a cross-reactive material 197 (CRM_197_) conjugate vaccine composed of the 6 most prevalent GBS CP serotypes, with potential coverage of 98% of GBS isolates causing neonatal invasive GBS disease, that is currently undergoing clinical evaluation (clinical trials registration NCT03170609).

## METHODS

### Serotype Determination

GBS isolates, obtained from the Tigecycline Evaluation and Surveillance Trial collection [16], were grown overnight, after which 200 μL was lysed with 10 µL of 5 U/µL mutanolysin (Sigma-Aldrich, St. Louis, MO) and 4 µL of 100 mg/mL lysozyme (ThermoFisher Scientific, Waltham, MA), and genomic DNA was purified (Agencourt Genfind V2 kit; Beckman Coulter, Beverly, MA). Primers and probes were designed against capsule type–specific sequences of types Ia, Ib, II, III, and V and *S. agalactiae*–specific *dltS* to allow detection by quantitative polymerase chain reaction analysis (Supplementary Tables 1 and 2); C_t_ values < 35 were considered to be positive results. GBS isolates that failed to yield a serotype by quantitative polymerase chain reaction analysis were serotyped using seroagglutination (catalog no. 54991; Statens Serum Institut, Copenhagen, Denmark). Any isolate failing to show agglutination within 30 seconds was designated “nontypable.” These strains were screened by sequencing for the presence of the CPS operon and assigned CPS types based on gene sequences from the *cpsG*-*cpsK* variable region of the CPS operon. Resulting sequences were submitted to GenBank (accession nos. MK402283–MK402291).

### Opsonophagocytic Activity (OPA) Assay

OPA assays that measured bacterial killing were developed with the following GBS strains of each capsular serotype: PFEGBST0779 (serotype Ia), PFEGBST0267 (Ib), PFEGBST0886 (II), PFEGBST0047 (III), PFEGBST0040 (IV), and PFEGBST0740 (V). Assays were performed as previously described [17], except for omission of a preincubation, and growth occurring at 30°C and 5% CO_2_, in 50% Todd-Hewitt medium with 0.5% yeast extract. The lower limit of detection equaled the lowest dilution tested (ie, 100 samples without a detectable OPA titer were assigned a value of 50).

### Detection of Anti-GBS Capsular Polysaccharide IgG and Immunoglobulin M (IgM) in Sera From Nonhuman Primates

A 6-plex direct Luminex immunoassay measured antibodies to each of 6 GBS CP serotypes. Serum samples were diluted in 50 μL assay buffer (phosphate-buffered saline containing 0.5% bovine serum albumin, 0.05% Tween-20, and 0.02% sodium azide; pH 7.2), mixed with an equal volume of 6-plex GBS CP–coated Luminex microspheres (50/serotype/μL), and incubated at 4°C for 20 hours. Following a washing step with assay buffer without bovine serum albumin, 50 μL of R-phycoerythrin–conjugated anti-human IgG or IgM secondary antibody (category nos. 109-115-098 and 709-116-073, respectively; Jackson Laboratories) were added, and plates were incubated for 90 minutes at room temperature, while shaking. After a final wash, 100 μL of washing buffer was added and fluorescence measured (catalog no. 171-000205; Bio-Plex, Biorad). Weight-based concentrations were calculated using an in-house reference standard serum, itself calibrated using standard human reference sera [18], and included on each assay plate. Results below the limit of quantification were assigned a value of half of the lower limit of quantification (LLOQ).

### Bioethics Regarding Animal Use

Animal studies were conducted according to Pfizer local and global institutional animal care and use committee guidelines at an Association for Assessment and Accreditation of Laboratory Animal Care International–accredited facility.

### Conjugates

Conjugates (in 5 mM succinate and 0.9% NaCl; pH 6.0) contained no significant endotoxin burden (<0.75 EU/µg conjugate). Samples of individual CP CRM_197_ conjugates were diluted in phosphate-buffered saline (with or without AlPO_4_) and combined to make GBS6.

### Statistical Analyses

Statistical differences of animal survival data were determined using a log-rank test (the Mantel-Cox test), and statistical differences of immunogenicity data were determined using log-transformed data followed by analysis by unpaired Student *t* tests with Welch correction of data (both are included in GraphPad Prism 7.02).

### Supplemental Information

More details on the experimental procedures are included in the Supplementary Materials.

## RESULTS

### GBS6 Design Based on GBS Epidemiology

**Figure 1.**
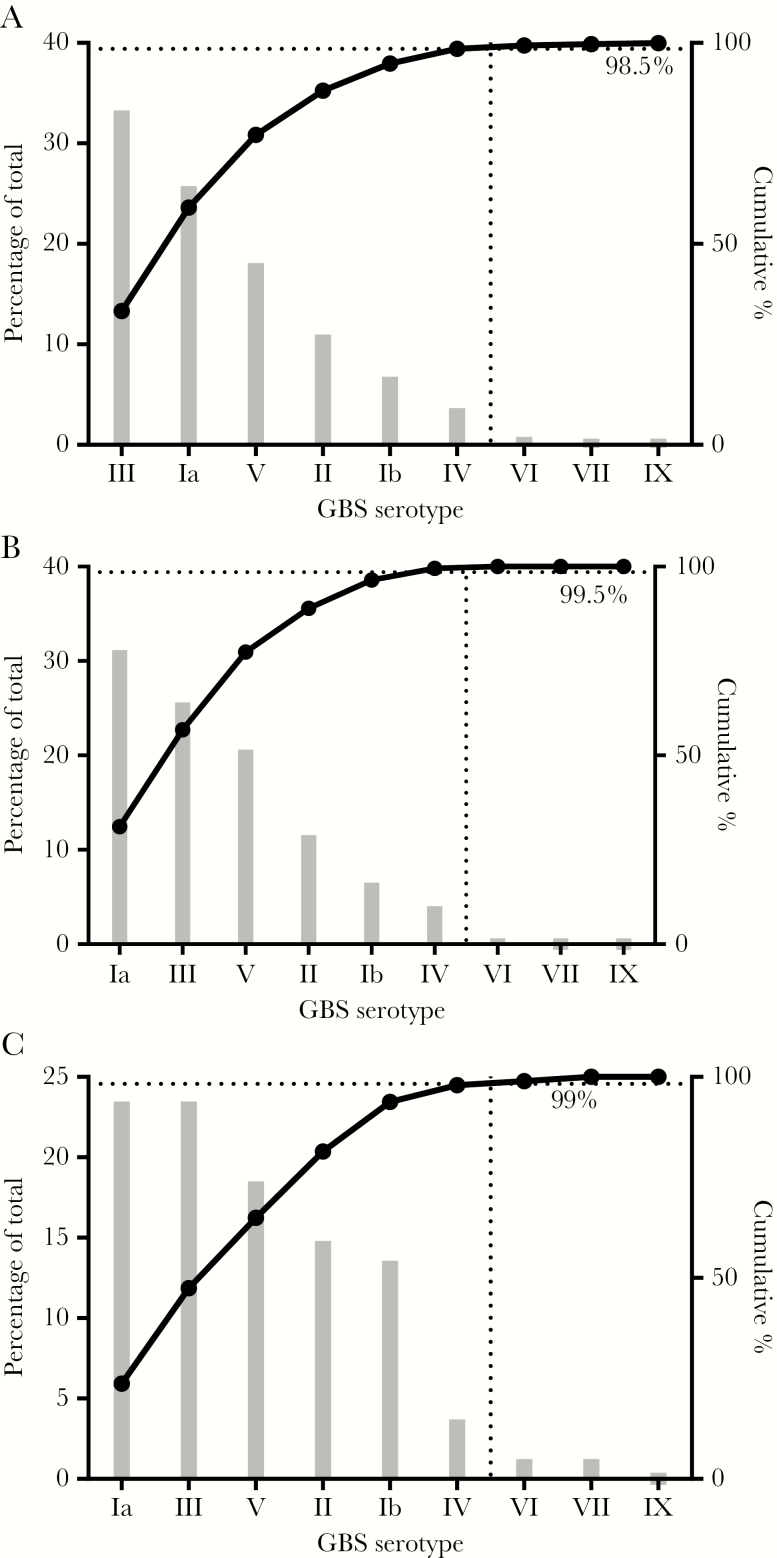
Prevalence of group B streptococcus (GBS) capsular polysaccharide (CP) serotypes. Strains were isolated globally from 901 infants with invasive disease (*A*), 199 infants with invasive disease in the United States (*B*), and 81 adults in the United States colonized by GBS (*C*). When combined, the GBS6 serotypes cover >98% of isolates in each collection.

Contemporary disease epidemiology was used to inform the vaccine design. CP serotypes were determined of a global collection of 901 GBS strains recently isolated from infants with invasive GBS disease [16] (Supplementary Table 3). Of these, 98% (888 of 901) belonged to one of 6 predominant serotypes Ia, Ib, II, III, IV, and V (Figure 1A and Supplementary Table 3). Analysis of a subset of 199 isolates from the United States provided a similar outcome except that the rank order of the most prevalent serotypes, Ia and III, was reversed (Figure 1A and 1B). As rectovaginal carriage is a risk factor for transmission of GBS to the infant, the study was expanded to include 81 carriage isolates obtained from the rectovaginal or perineal area of healthy US adult volunteers (Figure 1C). The main differences were the increased presence of serotype II (19 of 81 [23%]) and reduced presence of serotype III (11 of 81 [14%]) among carriage isolates. The identification that 6 serotypes were responsible for most disease justified the development of a hexavalent vaccine that included serotypes Ia, Ib, II, III, IV, and V.

### CP Sialylation Is a Key Quality Attribute for CP Conjugate Immunogenicity

**Figure 2.**
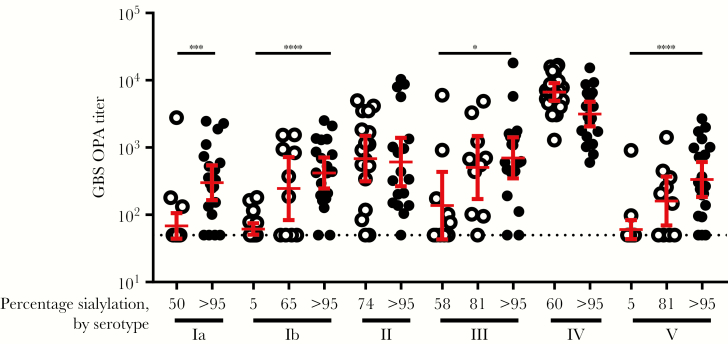
Sialic acid content of the capsular polysaccharide (CP) conjugates is critical for induction of functional immune responses. CP was either partially desialylated (open circles) or nontreated (closed circles) prior to conjugation. Groups of 10–20 female CD-1 mice were vaccinated with 1 μg of CP monovalent CP-CRM_197_ conjugate at weeks 0, 3, and 6. Serotype-specific opsonophagocytic activity (OPA) titers were determined in serum samples obtained at week 8. Geometric means are shown as bars with 95% confidence intervals. The lower limit of OPA detection was 100. The dotted line denotes a titer of 50, which was assigned to results below the limit of detection. Significant decreases after desialylation are shown. *P < .05, **P < .01, ***P < .001, and ****P < .0001.

Sialic acid moieties are the primary oxidation sites for GBS CP [19]. We evaluated their importance for inducing a functional immune response in mice by vaccination of conjugates prepared with sialylated and chemically desialylated polysaccharides [20]. A functional, bacterial killing response in sera was determined by OPA assays, rather than by measurement of CP binding antibodies that may not predict a functional antibody response [21]. For most serotypes, preserving high sialylation levels was confirmed to be critical to induce a strong immune response. Lowering of sialylation levels of serotype Ib and V to 5% significantly (*P* < .0001) reduced immunogenicity as compared to fully sialylated conjugates; for serotype Ia and III, even a smaller decrease to 50%–60% sialylation was detrimental (*P* < .05; Figure 2); results for serotypes II and IV were inconclusive: for serotype II, polysaccharides with low levels of sialylation could not be obtained, and for serotype IV, the assay was not sensitive enough to measure differences because high response rates were obtained for both conjugates. Therefore, high sialylation levels (typically, >95%) were retained in the vaccine polysaccharides; other critical quality attributes (Supplementary Table 4) were similar to those of other licensed conjugate vaccines, such as Prevnar 13 [22].

### Anti–GBS CP IgG Confers Maternal Protection in a Mouse Model

**Figure 3.**
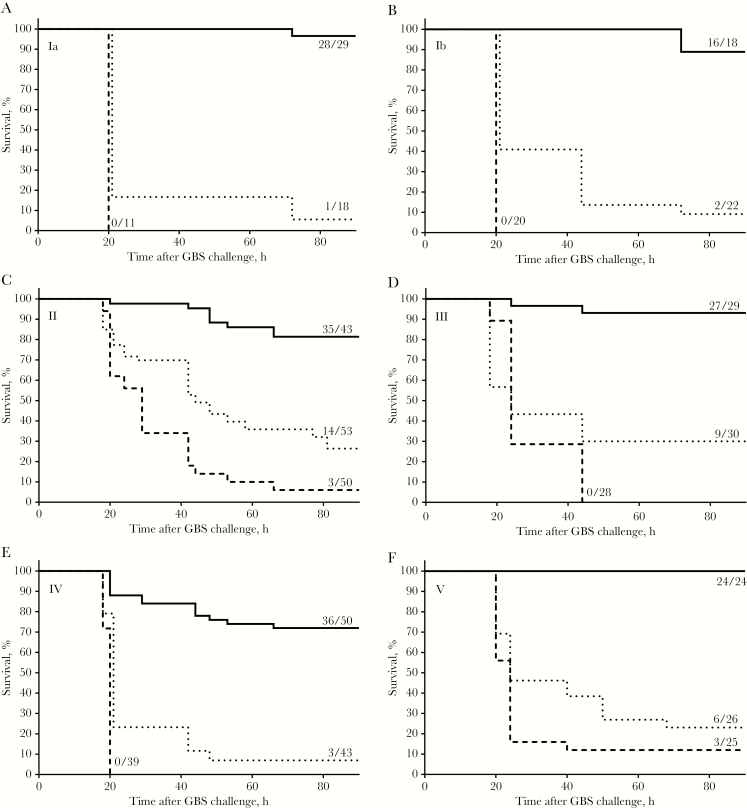
Passive immunization of dams with capsular polysaccharide-specific monoclonal antibody (mAb) protects pups against a lethal dose of group B streptococcus (GBS) bacteria of the corresponding serotype. Pups born to dams immunized 24–48 hours prior to delivery with phosphate-buffer saline (dotted line), 500 μg of unrelated immunoglobulin G (IgG) control mAb (dashed line), or 500 μg of capsular polysaccharide serotype–specific IgG mAb (straight line) were challenged within 24 hours after birth (day 0) with 10^5^–10^7^ colony-forming units, depending on serotype. Ratios indicate the number of surviving pups 90 hours after the challenge and the total number of pups tested. Protection by capsular polysaccharide–specific antibodies versus phosphate-buffered saline was statistically significant (*P* < .0001) for all serotypes.

To demonstrate preclinical efficacy and assess whether anti-CP IgG in a pregnant mouse (dam) is sufficient for protection of pups, a passive immunization model was developed using serotype-specific anti-CP monoclonal antibodies (mAb). Anti-CP serotype IgG1 mAb or an unrelated IgG1 mAb control was administered to dams 24–48 hours prior to delivery. Litters of 4–18 pups that were born to these dams were challenged within 24 hours after birth with 10^5^–10^7^ colony-forming units (depending on serotype) of a GBS strain of the corresponding serotype, reliably resulting in 5%–25% survival of pups born to unimmunized mice. After administration of serotype-specific IgG mAb, however, 72%–100% survival among pups was observed (Figure 3). Protection was thus demonstrated for mAb to each of the serotype capsules, indicating the placental transfer of serotype-specific anti-CP IgG to the pups and establishing that transfer of these antibodies alone is protective.

### GBS6 Protection in Mice

**Figure 4.**
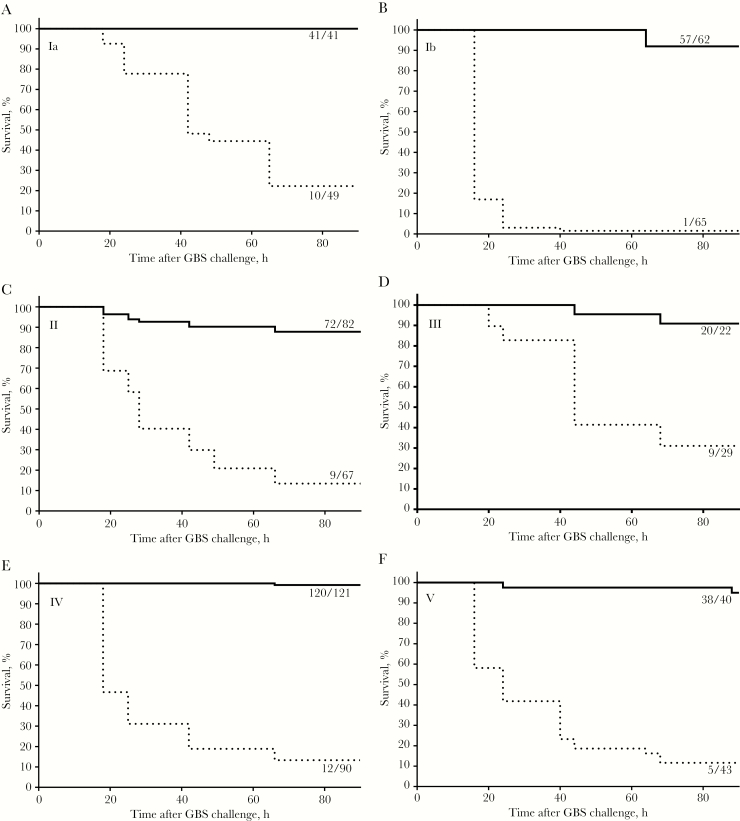
Active immunization of dams with GBS6 protects pups against a lethal dose of each of the 6 group B streptococcus (GBS) serotypes covered by the vaccine. Pups born to dams immunized 3 times with either phosphate-buffered saline (dotted line) or 5 µg of GBS6 (capsular polysaccharide/serotype; straight lines), each with AlPO_4_, were challenged within 24 hours after birth (day 0) with 10^5^–10^7^ colony-forming units, depending on the serotype. Ratios indicate the number of surviving pups 90 hours after the challenge and the total number of pups tested. Protection by vaccination with GBS6 versus phosphate-buffered saline was statistically significant (*P* < .0001) for all serotypes.

An active maternal immunization model [23] was used to evaluate protection of the pups resulting from vaccine-elicited immune responses in the dams. Female mice (2–10 per arm) were dosed at weeks 0, 3, and 6 with either 10 µg (CP/dose) of monovalent conjugate or 5 µg of GBS6 (CP/serotype/dose) formulated in 100 µL of 1 g/L AlPO_4_. Saline containing AlPO_4_ was used as a control. Mice were subsequently mated, and litters of 6–18 pups born to the dams were then challenged as described above. Pup survival after administration of saline control ranged from 1.5% to 31% (depending on serotype), which increased to 88%–100% after vaccination with GBS6 (Figure 4). Similar results were obtained after immunization with monovalent conjugates (Supplementary Figure 1). The data support the hypothesis that immunization with monovalent conjugates or GBS6 induced protective antibodies that were transferred to and protected the pups.

### GBS6 Immunization Induces Functional Antibodies in Rhesus Macaques

**Figure 5.**
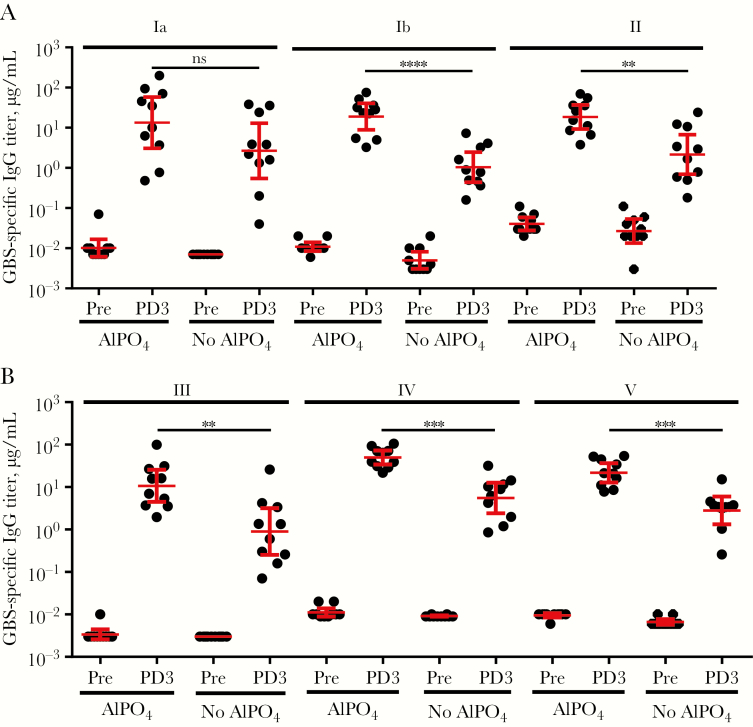
Immunoglobulin G (IgG) antibody response against all 6 group B streptococcus serotypes after administration of GBS6 to rhesus macaques. Groups of 10 rhesus macaques were vaccinated with 5 µg of GBS6 (capsular polysaccharide/serotype), with or without AlPO_4_, at weeks 0, 4, and 8. IgG concentrations were determined at week 0 (before vaccination) and week 10 (after delivery of dose 3). Geometric mean concentrations and 95% confidence intervals are shown as red bars. Increases from week 0 to 10 were all statistically significant (*P* < .0001; data not shown). NS, not significant. **P < .01, ***P < .001, and ****P < .0001.

**Figure 6.**
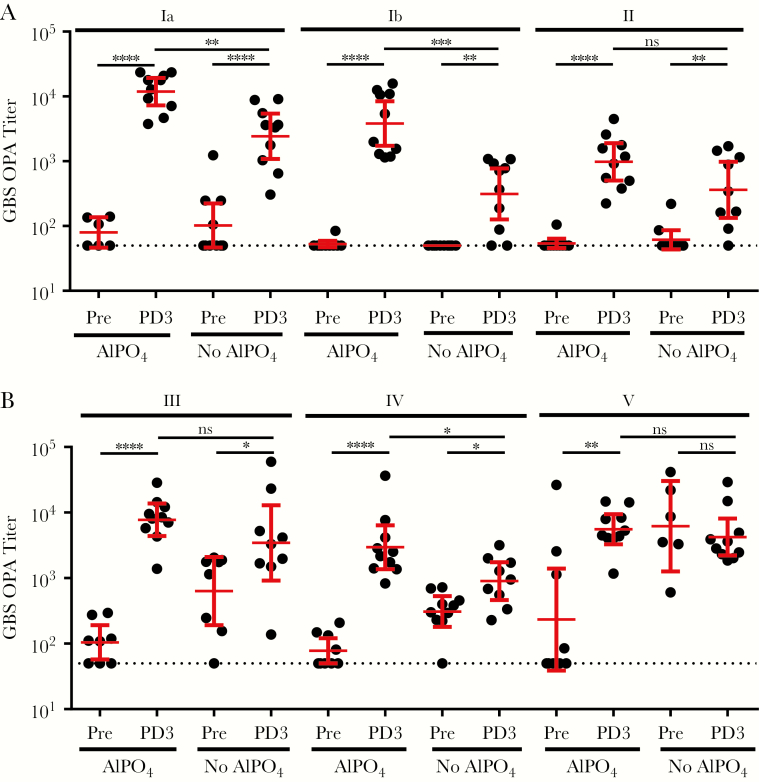
Functional antibody response, as measured by opsonophagocytic killing activity (OPA), against group B streptococcus (GBS) strains of each of 6 serotypes after administration of GBS6 to rhesus macaques. Groups of 10 rhesus macaques were vaccinated with 5 µg of GBS6 (capsular polysaccharide/serotype), with or without AlPO_4_, at weeks 0, 4, and 8. OPA titers were determined at week 0 (before vaccination) and week 10 (after delivery of dose 3). Geometric mean titers and 95% confidence intervals are shown as bars. The lower limit of OPA detection was 100. The dotted line denotes a titer of 50, which was assigned to results below the limit of detection. NS, not significant. *P < .05, **P < .01, ***P < .001, and ****P < .0001.

Although maternal anti-CP IgG concentrations are linked to protection against GBS disease in the newborn, CP conjugate vaccines can produce both IgG and IgM responses [24]. While anti-CP maternal IgM concentrations could protect the mother from maternal GBS disease, IgM cannot be transferred, thus rendering the induction of a strong IgG response by the pregnant mother key for protection of the baby [12]. In addition, it was critical to confirm that the formed anti-CP IgG was functional and mediated opsonophagocytic killing, the likely mechanism of action to protect against GBS disease. To assess an optimal GBS6 formulation, the potential value of adding of AlPO_4_ [25] was also evaluated. Rhesus macaques were immunized with 5 µg of GBS6 (CP/serotype/dose) formulated with or without AlPO_4_ (0.5 mg/mL) in a 1-mL volume. The monkeys were immunized at weeks 0, 4, and 8, with blood specimens collected at weeks 0 and 10. GBS6 serotype–specific IgG concentrations and OPA titers in sera from nonimmunized rhesus macaques were generally low, with the exception of the OPA titers against serotype V in the group that received nonadjuvanted vaccine (Figures 5 and 6). After vaccination with GBS6, however, high anti-CP IgG responses (Figure 5) and functional, opsonophagocytic killing activity (Figure 6) developed against all 6 serotypes, particularly when formulated with AlPO_4_. Measurable preimmunization IgM serum concentrations were present in all rhesus monkeys, with geometric mean concentrations (GMCs) of 1–10 μg/mL at baseline increasing by up to an order of magnitude following vaccination (Supplementary Figure 2). Although GBS6 IgM concentrations observed at baseline appeared elevated, the low OPA activity in these sera suggested that these antibodies had limited functional, bacterial killing activity. In contrast, anti-CP IgG serum GMCs were <0.01 μg/mL at baseline but increased to 0.9–5.5 μg/mL after administration of GBS6 without AlPO_4_ (Figure 5). In general, the vaccine-induced increase in OPA response correlated with the vaccine-induced increase of anti-CP IgG binding antibodies. Inclusion of AlPO_4_ augmented the anti-CP IgG concentrations to 11–50 μg/mL, yielding statistically significant increases (*P* < .05) for 5 of 6 serotypes, compared with the non-AlPO_4_ formulations.

## DISCUSSION

Shortly after the protective effect of maternal GBS anti-CP IgG antibodies was recognized [9], efforts to develop a vaccine followed, culminating in testing of the safety and immunogenicity of serotype Ia, Ib, II, III, and V CPs individually conjugated to tetanus toxoid in human [14, 24, 26–28]. Although these monovalent vaccines were demonstrated to be safe and immunogenic in pregnant and nonpregnant women, further advancement to late-stage clinical testing was halted for 2 reasons. First, coincident with the attempted development of a vaccine, guidance for GBS carriage screening during pregnancy and subsequent intrapartum antibiotic prophylaxis for colonized mothers were introduced, which reduced the rate of early onset disease in the United States [4] and thereby diminished the perceived need for a GBS vaccine. Second, manufacturers were cautious about the development of maternal vaccines, given that there was and still is no precedent for a vaccine licensed for maternal use. More recently, however, a number of factors changed that encouraged the development of vaccines for maternal use. While intrapartum antibiotic prophylaxis has been effective at reducing early onset disease, remaining rates are still substantial, and such prophylaxis has not decreased rates of late-onset disease [29]. Furthermore, the continuous emergence of novel influenza virus strains and the resurgence of pertussis have led to the recommendation of influenza and tetanus/diphtheria/acellular pertussis vaccines for US women during each pregnancy [30, 31]. The substantial health benefit to the infant and the pregnant mother, combined with a more robust safety database for maternal immunization, have established a precedent for vaccinating pregnant women and lowered the hurdle to develop a maternal vaccine such as GBS6.

The relative prevalence of specific CP serotypes in a global collection of isolates obtained from invasive neonatal disease cases was critical to determining the optimal serotype valence to include in the vaccine, because the data demonstrated the dynamic nature of GBS (Figure 1). Whereas in the mid-1970s only serotypes Ia, Ib, II, and III were found among invasive neonatal isolates [32], by the end of the century serotype V had emerged [33]. Our data also confirm the emergence of serotype IV in the United States [34] and extend this observation as a global trend (Supplementary Table 3). This rise has been proposed to be due to large recombination events that introduced the type IV capsule operon and replaced the type Ia and III *cps* loci in the more virulent CC23 and CC17 strains, respectively [35]. A limitation to our analysis was the small number of isolates assessed from Asia (n = 12); however, our finding that a CP vaccine containing 6 serotypes (Ia, Ib, II, III, IV, and V) has the potential to provide high coverage is corroborated by findings from recent studies conducted in Asia [36–40].

Efficacious maternal vaccines need to induce functional IgG antibodies because these are uniquely transferred from the mother to the fetus through the placenta [12]. Because GBS CP vaccines resulted in a poor immune response [13], CP conjugate vaccines were preferentially developed for their ability to drive CD4^+^ T-helper cells, leading to induction of immune memory with predominantly IgG, as opposed to IgM, antibody responses [41]. Because GBS6 is being developed for pregnant women, its safety and tolerability are particularly important. Therefore, CRM_197_ was selected as a carrier protein because it has been safely administered to hundreds of millions of infants and adults as a component of pneumococcal and meningococcal conjugate vaccines [25, 42]. CRM_197_ was also used for the investigational GBS3 vaccine in pregnant women, with no reported safety concerns for the mother or infant [43, 44] despite observations that the use of a carrier that is used in other vaccines may lead to a phenomenon known as carrier suppression, in which subsequent immune responses can be reduced [45]. This will be assessed during the GBS6 clinical program. In GBS3 clinical studies, no overall benefit was observed for the overall IgG responses when Al(OH)_3_ was coadministered. Our data in nonhuman primates show that AlPO_4_ may provide a benefit and augment serotype-specific IgG concentrations (Figure 5) and the quality of the functional immune response (Figure 6), justifying the evaluation of GBS6 with AlPO_4_ in humans. Furthermore, the inclusion of AlPO_4_ in the pneumococcal conjugate vaccines has been shown for at least some serotypes to direct improved induction of IgG1 [46], which is the preferred IgG subclass for the transfer of protective antibodies across the placenta [12].

A number of seroepidemiology studies have proposed various serotype-specific maternal IgG serum concentrations as serological correlates of protection against early onset disease and late-onset disease occurring in the first month of life, caused by prevalent serotypes Ia and III [14]. Although standardized assays to monitor GBS6 immune responses are not yet available, the most recent serological studies, as well as the work described here, used Dr Carol Baker’s standardized human reference sera in the assays [18], allowing for an initial comparison. Proposed correlates range from 0.5 to 2 µg/mL for early onset disease and from 0.5 to 6 µg/mL when late-onset disease was included [14]. All conjugates in GBS6 were immunogenic in rhesus macaques, leading to high serum GBS6 IgG concentrations (Figure 4) and killing OPA responses (Figure 6). The protective thresholds were typically exceeded in rhesus macaques, with serotype-specific IgG GMCs ranging from 11 to 50 μg/mL when AlPO_4_ was included and often even when GBS6 was administered without adjuvant (GMC range, 0.9–5.5 μg/mL; Figure 5). It should be pointed out that, whereas the monkeys were immunized 3 times, humans, unlike nonhuman primates, are already primed by GBS carriage. Therefore, a single administration of GBS6 may likely suffice to induce an anamnestic response adequate enough to protect the pregnant mother and baby during time of delivery.

The anti-CP IgG responses against all serotypes were virtually identical (Figure 5) and did not confirm the previously reported high serotype V–specific IgM response, relative to that of IgG, that led to the suggested remediation by using desialylated serotype V CP [24, 47]. Here, we showed that preservation of sialylation of serotype V conjugates was critical for a functional OPA response in mice (Figure 2) that was transferred at protective levels to the pup (Figure 4). As Gutthormsen et al suggest, the CP/protein ratios of conjugates may be an important cause of the difference in response [47]. Whereas in their studies the ratio ranged from 2.3–3.2 (w/w) for various sialylated serotype V conjugates, those of their desialylated serotype V conjugate and sialylated serotype II and III conjugates, which yielded the desired predominantly IgG response, had ratios of 0.7–1.3 (w/w), very similar to ratios for each of the optimized conjugates presented in part here (Supplementary Table 4). The functional immune responses to conjugates of each of the serotypes described here were also very similar. The exception was the lack of increase in the functional response to serotype V when GBS6 without AlPO_4_ was administered (Figure 6), whereas a strong IgG response was observed (Figure 4). This also strongly contrasts with the functional response seen in non-human primates in the presence of AlPO_4_ (Supplementary Table 4) and the protection of the pups born to dams immunized with serotype V conjugates from a lethal GBS challenge (Figure 4 and Supplementary Figure 1). At this point, it is unclear why the serotype V functional response at baseline was so high and why no additional increase in that response was observed in the GBS6 formulation without AlPO_4_.

In summary, GBS6 was designed to potentially cover 98% of GBS isolates causing neonatal invasive GBS disease. Preclinical proof-of-concept studies demonstrated protection of pups born to vaccinated mice and a robust IgG-mediated functional immune response in rhesus macaques that was enhanced by inclusion of AlPO_4_ adjuvant. Therefore, it is anticipated that vaccination of pregnant women with GBS6 will elicit strong functional and protective IgG responses, which will be transferred to their infants prior to birth. Ongoing clinical studies in nonpregnant volunteers (clinical trials registration NCT03170609) are required to evaluate translation of the preclinical promise of GBS6 into clinical safety and immunogenicity and will serve as a critical stepping stone to ultimately address the severe unmet medical need for the prevention of early onset and late-onset GBS disease in infants globally.

## Supplementary Data

Supplementary materials are available at The *Journal of Infectious Diseases* online. Consisting of data provided by the authors to benefit the reader, the posted materials are not copyedited and are the sole responsibility of the authors, so questions or comments should be addressed to the corresponding author.

jiz062_suppl_Supplementary_Material

## Notes

***Acknowledgments.* ** We thank the following Pfizer employees: Arthur Illenberger and Jennifer Obregon, for assistance with rhesus macaques; Li Hao, Lubomira Andrew, Bihui Xu, and Lorna Nunez, for WGS analyses; Hal Jones and Eneida Pollozi, for microbiology support; Peimin Lu and Julio Hawkins, for OPA analyses; and Erik Johnson and Isis Kanevsky, for critical reading of the manuscript.

***Financial support.* **This work was supported by Pfizer.

***Potential conflicts of interest.* **All authors are employees of Pfizer and may, as a consequence, be shareholders. All authors have submitted the ICMJE Form for Disclosure of Potential Conflicts of Interest. Conflicts that the editors consider relevant to the content of the manuscript have been disclosed.
