# Supplementary material for: A Novel Hexavalent Capsular Polysaccharide Conjugate Vaccine (GBS6) for the Prevention of Neonatal Group B Streptococcal Infections by Maternal Immunization
Source: J Infect Dis. 2019 Feb 19;220(1):105–15. doi: 10.1093/infdis/jiz062 (PMC6548902; doi:10.1093/infdis/jiz062)
Supplement: jiz062_suppl_Supplementary_Material [file jiz062_suppl_supplementary_material.docx]

**Supplementary Information**

A novel hexavalent capsular polysaccharide conjugate vaccine (GBS6) for the prevention of neonatal group B streptococcal infections by maternal immunization

**Ed T. Buurman, Yekaterina Timofeyeva, Jianxin Gu, Jin-hwan Kim, Srinivas Kodali, Yongdong Liu, Terri Mininni, Soraya Moghazeh, Danka Pavliakova, Christine Singer, Suddham Singh, Luke D. Handke, Jason Lotvin, A. Krishna Prasad, Ingrid L. Scully, Robert G. K. Donald, Kathrin U. Jansen, and Annaliesa S. Anderson**

| Supplemental Table S1. qPCR assay primers for detection of GBS serotypes Ia, Ib, II, III and V. | | |
| --- | --- | --- |
| Name | Oligonucleotide sequence | Description |
| oLH616 | GTG GCC TGC TGG GAT TAA TG | Forward *cpsH* primer serotype Ia |
| oLH618 | AGA TCT TCA AGT GCA AAA AAT GAG AA | Reverse *cpsH* primer serotype Ia |
| oLH619 | TGA ACA AAG CGG TGA AAA TTT CT | Forward *cpsJ* primer serotype Ib |
| oLH621 | TTT CCT CCA TAA AAC GAA TTT GC | Reverse *cpsJ* primer serotype Ib |
| oLH622 | CCC GAT ATC ATG GCT TCA GTA AG | Forward *cpsK* primer serotype II |
| oLH623 | TCT CTC ATC TGT TGG TAA ATC TTT CAA | Reverse *cpsK* primer serotype II |
| oLH626 | TGG CGC TAG AAT ACT ATT GGT CTG T | Forward *cpsH* primer serotype III |
| oLH627 | AAC AAG CGG TGA TAA ATA AGA AAG TCA | Reverse *cpsH* primer serotype III |
| oLH634 | TGA GTG TTT TCT AGC AAG GGA GAT T | Forward *cpsM* primer serotype V |
| oLH635 | TGT CCT TTA ACA GCG CCA ATT | Reverse *cpsM* primer serotype V |
| oLH640 | TTT AGG AAT ACC AGG CGA TGA AC | Forward *dltS* primer GBS |
| oLH641 | GCT TTG AAT CTT AAC CAT CTT TTG G | Reverse *dltsS* primer GBS |

| Supplemental Table S2. Probes used in the qPCR assay. | | | | |
| --- | --- | --- | --- | --- |
| Probe name | Probe sequence | 5’ modification^a^ | 3’ modification^b^ | Description |
| prLH2 | TTA CTA ATA TTG GTA GGA AAG CT | 6-FAM | MGBNFQ | *cpsH* gene probe specific for GBS serotype Ia |
| prLH3 | TTT TTA GAA TGG ATG CTG ATG AT | 6-FAM | MGBNFQ | *cpsJ* gene probe specific for GBS serotype Ib |
| prLH5 | ATT GTA AGA CGA TAG AAG TAA G | 6-FAM | MGBNFQ | *cpsK* gene probe specific for GBS serotype II |
| prLH15 | TTG ACC AAG AAA AAC ACT TT | VIC | MGBNFQ | *cpsH* gene probe specific for GBS serotype III |
| prLH16 | TTG TGA TGT GAA TAC AGG ATT | VIC | MGBNFQ | *cpsM* gene probe specific for GBS serotype V |
| prLH17 | ATT GCT TTG GTG ACT ATA G | VIC | MGBNFQ | Probe specific for GBS *dltS* |
| ^a^6-FAM = 6-fluorescein amidite, VIC = VIC fluorescent dye  ^b^MGBNFQ = minor groove binder/nonfluorescent quencher | | | | |

| Supplemental Table S3. Geographic distribution of GBS isolates obtained from neonates with invasive disease (2004-2013) | | | | | | | | | | |
| --- | --- | --- | --- | --- | --- | --- | --- | --- | --- | --- |
|  |  | Percentage Serotype Prevalence (n) | | | | | | | | |
| **Region** | **Total** | **Ia** | **Ib** | **II** | **III** | **IV** | **V** | **VI** | **VII** | **IX** |
| North America | 208 | 31.3 (65) | 6.7 (14) | 11.5 (24) | 25.0 (52) | 3.8 (8) | 21.1 (44) | 0.5 (1) | 0 | 0 |
| South America | 56 | 28.6 (16) | 8.9 (5) | 14.3 (8) | 33.9 (19) | 0 | 14.3 (8) | 0 | 0 | 0 |
| Europe | 565 | 23.2 (131) | 6.9 (39) | 10.5 (59) | 35.9 (203) | 4.1 (23) | 18.4 (104) | 0 | 0.5 (3) | 0.5 (3) |
| Africa | 22 | 40.9 (9) | 0 | 9.1 (2) | 45.5 (10) | 0 | 4.5 (1) | 0 | 0 | 0 |
| Middle East | 18 | 16.7 (3) | 11.1 (2) | 16.7 (3) | 27.7 (5) | 11.1 (2) | 16.7 (3) | 0 | 0 | 0 |
| South Pacific | 20 | 20.0 (4) | 5.0 (1) | 15.0 (3) | 40.0 (8) | 0 | 10.0 (2) | 10.0 (2) | 0 | 0 |
| Asia | 12 | 33.3 (4) | 0 | 0 | 25.0 (3) | 0 | 8.3 (1) | 33.3 (4) | 0 | 0 |
| Global | 901 | 25.7 (232) | 6.8 (61) | 11.0 (99) | 33.3 (300) | 3.7 (33) | 18.1 (163) | 0.8 (7) | 0.3 (3) | 0.3 (3) |

| **Supplemental Table S4. Characterization of GBS6 CRM_197_ conjugates** | | | | | | |
| --- | --- | --- | --- | --- | --- | --- |
|  | Serotype | | | | | |
| Property | Ia | Ib | II | III | IV | V |
| MW CP^1^ (kDa)^2^ | 190 | 120 | 95 | 263 | 143 | 132 |
| Sialic acid content CP (%)^3^ | >95 | >95 | >95 | >95 | >95 | >95 |
| CP modification (%)^4^ | 6 | 6 | 8 | 10 | 7 | 7 |
| CP/protein ratio (w/w) | 0.8 | 1.1 | 0.84 | 1.1 | 0.80 | 1.32 |
| % Free CP (mol/mol) | <5 | 11 | 16 | 10 | <5 | 11 |
| MW conjugate (kDa)^2^ | 6040 | 2608 | 3600 | 2396 | 8268 | 4304 |
| OPA (GMT)^5^ | 300 | 417 | 610 | 701 | 3140 | 335 |
| ^1^Capsular polysaccharide; ^2^SEC-MALS; ^3^Mol per repeat unit; ^4^Mol repeat unit/mol aldehyde; ^5^Opsonophagocytic Activity (Geomean titer) | | | | | | |

**Supplemental Fig. S1.** Active immunization of dams with monovalent capsular polysaccharide CRM_197_ conjugate protects pups against a lethal dose of GBS bacteria of the corresponding serotype. Pups born to dams immunized three times with either phosphate-buffered saline (dotted line), or 10 µg monovalent CRM_197_-conjugate (straight line), each with AlPO_4_, were challenged within 24 hrs after birth (day 0) with 10^5^-10^7^ CFU, depending on the serotype. Ratios indicate the number of surviving pups 90 hrs after the challenge and the total number of pups tested. Protection by vaccination with monovalent conjugates vs. phosphate-buffered saline was statistically significant (p<0.0001) for all serotypes.

**Supplemental Fig. S2.** IgM antibody response against all six serotypes after administration of GBS6 to Rhesus macaques. Groups of Rhesus macaques (n=10) were vaccinated with 5 µg GBS6, with or without AlPO_4_, at Weeks 0, 4 and 8. IgM concentrations (µg/mL) were determined at Week 0 (PRE) and Week 10 (post dose (PD3)). Geometric means and 95% CI are shown as bars. Statistical significance: Ns: not significant, *p<0.05 **p<0.01 ***p<0.001 ****p<0.0001.

**Determination of serotypes of GBS isolates.** Individual colonies from blood agar plates (Becton Dickinson, Franklin Lakes, NJ) were resuspended into individual wells of a 96-well deep-well plate containing 1 mL of Todd Hewitt broth + 0.5% yeast extract medium (THY) (Becton Dickinson), after which the plate was sealed with a gas-permeable cover and the plate was incubated overnight at 37°C without shaking in a 5% CO_2_ incubator. The following day, the cells were mixed by pipetting, and 200 μL transferred to a Greiner PP-Masterblock 96-well 2 mL deep-well plate (Greiner Bio-One, Monroe, NC) containing 14 μL lysis solution in each well (10 µL 5 U/µL mutanolysin [Sigma-Aldrich, St. Louis, MO], 4 µL 100 mg/mL lysozyme [ThermoFisher Scientific, Waltham, MA]). A sterile foil cover was placed on the plate, and the plate was incubated at 37°C for 30-60 min.  Contents were lysed and genomic DNA purified as described for the Agencourt GENFIND V2 kit (Beckman Coulter, Beverly, MA). Genomic DNA from these plates, and control plates containing known quantities of genomic DNA from *S. agalactiae* capsule type isolates, were diluted 1:5,000 in sterile water with a PlateMate 2x3 robot (ThermoFisher Scientific).

A 384-well Taqman qPCR assay was developed for identification of GBS capsule types Ia, Ib, II, III, and V. Capsule type-specific sequences were selected using previously identified restriction fragment length polymorphisms as a guide (Manning et al. 2005), and nucleotide sequence alignments of reference CP gene sequences available from GenBank. The specificity of the resulting primer-probe sets was confirmed by PCR against a panel of GBS isolates of known capsule type. Sequences of primers (Integrated DNA Technologies, Coralville, IA) and probes (Life Technologies, Carlsbad, CA) are listed in Supplemental Table S1-2. A PCR amplicon specific for the *S. agalactiae*-specific gene, *dltS* (Poyart et al 2001), was incorporated into the assay to confirm correct isolate speciation and the integrity of the purified genomic DNA. The *dltS* primer-probe set was shown to be specific for GBS when tested against a panel of streptococcal and enterococcal species (data not shown). A duplex qPCR format was developed in which reactions specific for capsule type Ia and *dltS*, for capsule type Ib and capsule type V, and for capsule type II and capsule type III were combined.

A volume of 15 mL of qPCR master mix (Supplemental Table S3) was added to each reservoir of an Axygen Low Profile 12 channel trough plate (Corning, Corning, NY), and the plate was tilted back and forth to cover each trough evenly. A PlateMate 2x3 robot (ThermoFisher Scientific) was used to dispense 6 μL of master mix to each well of an Agilent 384-well optical plate (Agilent Technologies, Santa Clara, CA). The mixes yielded five 384-well plates for each primer-probe set that were covered with sterile foil covers and kept in the dark at 4°C until use. A volume of 4 µL of genomic DNA was added to each well in the reaction plate after which PCR reactions were run in an ABI 7900HT instrument (Applied Biosystems, Foster City, CA) with an initial denaturation for 3 min at 95°C, followed by 40 cycles of 95°C for 3 seconds and 60°C for 35 seconds. Data collection was performed during the 60°C step, and C_t_ values were determined following analysis with SDS 2.3 software (Applied Biosystems). Isolates were identified as *S. agalactiae* if the C_t_ value with the *dltS*-specific primer-probe set was < 35. Isolates were assigned a capsule type if the C_t_ for a capsule type-specific primer-probe set was < 35.

GBS isolates that failed to yield a serotype by qPCR genotypic analysis were serotyped using a kit (Statens Serum Institut, Copenhagen, Denmark; cat# 54991). Serotype-specific antibodies coupled to blue latex beads were mixed with a suspension of bacteria in PBS buffer and the seroagglutination reaction scored. A drop (~10 μL) of latex bead-antibody suspension was combined with an equal volume of bacterial sample on a white reaction card (Statens Serum Institut; cat# 53285). Bacterial suspensions were prepared by scraping a loopful of colonies from an overnight blood-agar plate culture and resuspended to an OD_600_ of >1.0 in PBS buffer. If clumping of the latex beads occurred within 30 sec, a positive result was recorded and serotype assigned.

Isolates that failed to genotype by qPCR or serotype by latex bead agglutination were subject to genomic sequencing with the Illumina Miseq platform. CLC workbench software was used to generate *de novo* genome assemblies from individual DNA sequence reads to identify the capsular polysaccharide operons. The BIGSdb open source platform (Jolley & Maiden 2010) was used to store, retrieve, and further analyze genotypic and associated phenotypic information. A bioinformatic BLAST algorithm based on reference gene sequences from the *cpsG*-*cpsK* variable region that are specific for each serotype was used to scan the CPS operon of each strain for identity. Only nine of the 901 neonatal isolates required genome sequence analysis to resolve their non-typable status. Sequences of the CPS biosynthetic gene cluster spanning *cpsR* through *neuA* genes in these strains were extracted (GenBank MK402283-MK402291) and aligned with serotype-specific *cps* operon reference sequences to confirm serotype assigned by BLAST algorithm. Strain PFEGBS0684 was a CPS phenotypic null mutant and could not be serotyped *in silico* due to a 10kb deletion in *cpsF* through *neuA* genes. The remaining eight strains were assigned a serotype based on the presence of intact *cps* operon sequences: three serotype IV strains (PFEGBST0557, PFEGBST0415 and PFEGBST0711) were found to be defective in CPS expression either by sialic acid HPLC assay or by flow cytometry with serotype-specific antibodies (data not shown), and harbor potential *cps* mutations based on comparisons with reference operon sequences (e.g. accession number LT671987.1); five other strains expressed serotype IV CPS (PFEGBST0122) or serotype Ia CPS (PFEGBST0696, PFEGBST0509, PFEGBST0673 and PFEGBST0859) and were presumably mistyped during initial qPCR genotyping and latex bead seroagglutination.

**Opsonophagocytic activity (OPA) assay.** A well characterized OPA assay monitoring bacterial killing was used, based on that developed for *Streptococcus pneumoniae* (Cooper et al 2011) with the following modifications. GBS strains representative of each capsular serotype, PFEGBST0779 (Ia), PFEGBST0267 (Ib), PFEGBST0886 (II), PFEGBST0047 (III), PFEGBST0040 (IV) and PFEGBST0740 (V), were obtained from the invasive clinical strain collection. Heat-treated test sera were two-fold serially diluted to which differentiated HL-60 cells (~400,000/well), baby rabbit complement (10% (v/v)) (PelFreez Biologicals, Rogers, AR) and bacterial cells (~2000 CFU/well) were added, leaving out a preincubation step, to a total volume of 100 µL. The plate was incubated for 60 min at 30°C and 5% CO_2_, while shaking at 400 rpm. Assay mixtures were filtered through prewashed 96-well Millipore filter plates (Cat # S5EJ085109), followed by 50% Todd-Hewitt medium with 0.5% yeast extract. After overnight incubation, plates were fixed, stained with QC Colloidal Coomassie Stain (BioRad, Hercules, CA), and destained. GBS colonies were enumerated with an ImmunoSpot plate reader (Cellular Technology Ltd, Cleveland, OH). The titer was expressed as the reciprocal of the lowest dilution of serum yielding a 50% reduction in colony forming units (CFU) versus the no-serum control. The lower limit of detection (LLOD) was equal to the lowest dilution tested, i.e. 100; samples that did not have a detectable OPA titer were assigned a value of 50.

**Detection of anti-GBS capsular polysaccharide IgG and IgM in sera from non-human primates.** A 6-plex GBS direct Luminex Immunoassay (dLIA) was based on Luminex MagPlex® xMAP® technology platform and measured antibodies to each of six GBS capsular polysaccharide serotypes (Ia, Ib, II, III, IV and V) (Pickering et al 2002). GBS capsular polysaccharides of each serotype were individually conjugated to poly-L-lysine and, in turn, coupled to spectrally distinct polystyrene superparamagnetic, carboxylated microspheres for each of the six antigens. All test and reference serum samples were diluted in assay buffer (phosphate-buffered saline (PBS) containing 0.5% BSA, 0.05% Tween-20, 0.02% sodium azide, pH 7.2). Each sample was tested in duplicate, starting with a 500-fold dilution and then three five-fold serially dilutions for three data points. A volume of 50 μL of diluted sera was transferred to wells of a 96-well flat bottom opaque white plate (Costar #3912, Corning Inc, Corning NY) and an equal volume of 6-plexGBS PS-coated Luminex microspheres (50/serotype/μL) was added. Plates were sealed and incubated at 4^o^C for 20 hrs. Following a washing step with washing buffer (assay buffer from which BSA was omitted) to remove non-bound antibodies, 50 μL of R-phycoerythrin-conjugated anti-human IgG or IgM secondary antibody (Jackson Laboratories, cat #109-115-098 and 709-116-073, respectively) were added to the wells, to detect either primate or human serum IgG or IgM antibodies. Sealed plates were incubated for 90 min at room temperature, while shaking. After a final wash, 100 μL washing buffer was added and the fluorescent R-phycoerythrin signal was measured in a Bio-Plex 200 Reader (Biorad, #171-000205). Weight-based antibody concentrations (μg/mL) for the unknown test samples and quality control samples were calculated using in-house reference standard serum that itself was calibrated using standard human reference sera (Pannaraj et al 2009), and included on each assay plate. Results below the limit of quantification (BLQ) were assigned a value of ½ of the lower limit of quantification (LLOQ).

**Monoclonal antibodies**. Hybridomas were generated from a fusion of a nonsecreting myeloma cell line (X63Ag8.653, ATCC) with splenocytes that were harvested from mice immunized with monovalent GBS CPS-CRM197 conjugate. Anti-GBS CPS mAb–secreting hybridomas were selected with the corresponding serotype conjugate and counter-selected with the other five serotype conjugates in enzyme-linked immunosorbant assays using standard procedures, and flow cytometry.

References

1. Brandon M, Dowzicky MJ (2013) Antimicrobial susceptibility among Gram-positive organisms collected from pediatric patients globally between 2004 and 2011: results from the Tigecycline Evaluation and Surveillance Trial. *J Clin Microbiol* 51(7):2371-2378.
2. Cooper D, et al. (2011) The 13-valent pneumococcal conjugate vaccine (PCV13) elicits cross-functional opsonophagocytic killing responses in humans to *Streptococcus pneumoniae* serotypes 6C and 7A. *Vaccine* 29(41):7207-7211.
3. Jolley KA, Maiden MC (2010) BIGSdb: Scalable analysis of bacterial genome variation at the population level. *BMC Bioinformatics* 11:595
4. Manning SD, et al. (2005) DNA polymorphism and molecular subtyping of the capsular gene cluster of group B streptococcus. *J Clin Microbiol* 43(12):6113-6116.
5. Pannaraj PS, et al. (2009) Group B streptococcal conjugate vaccines elicit functional antibodies independent of strain O-acetylation.*Vaccine* 27(33):4452-6
6. Pickering JW, et al. (2002) A multiplexed fluorescent microsphere immunoassay for antibodies to pneumococcal capsular polysaccharides. *Am J Clin Pathol* 117(4):589-596.
7. Poyart C, et al. (2001) Regulation of D-alanyl-lipoteichoic acid biosynthesis in *Streptococcus agalactiae* involves a novel two-component regulatory system. *J Bacteriol* 183(21):6324-6334.
